# *Oroxylum indicum* (L.) Kurz extract inhibits adipogenesis and lipase activity in vitro

**DOI:** 10.1186/s12906-018-2244-3

**Published:** 2018-06-08

**Authors:** Tanaporn Hengpratom, Gordon M. Lowe, Kanjana Thumanu, Siriporn Suknasang, Kanokwan Tiamyom, Griangsak Eumkeb

**Affiliations:** 1 School of Preclinic, Institute of Science, Suranaree University of Technology, Nakhon Ratchasima, 3000 Thailand; 2 School of Pharmacy and Biomolecular Sciences, Liverpool John Moores Univerisity, James Parsons Building, Byrom Street, Liverpool, L3 3AF UK; 3 Synchrotron Light Research Institute (Public Organization), Suranaree Subdistrict, Muang District, Nakhon Ratchasima, 30000 Thailand

**Keywords:** *Oroxylum indicum* (L.) Kurz, Adipogenesis, Lipase activity, 3T3-L1 adipocytes, FTIR microspectroscopy, Lipid-lowering drugs

## Abstract

**Background:**

*Oroxylum indicum* (L.) Kurz (*O. indicum*) is found in Thailand. It has been used for the treatment of obesity. This study aimed to investigate the effects of an *O. indicum* extract (OIE) on the adipogenic and biomolecular change in 3T3-L1 adipocytes.

**Methods:**

Initial studies examined the chemical components of OIE. The cell line 3T3-L1 was used to establish potential toxic effects of OIE during the differentiation of pre-adipocytes to adipocytes. The inhibitory effect of OIE on lipid accumulation in 3T3-L1 cells was investigated. Moreover, the impact of OIE on pancreatic lipase activity was determined. In further experiments, Fourier Transform Infrared (FTIR) was used to monitor and discriminate biomolecular changes caused by the potential anti-adipogenic effect of OIE on 3T3-L1 cells.

**Results:**

Chemical screening methods indicated that OIE was composed of flavonoids, alkaloids, steroids, glycosides, and tannins. The percentage viability of 3T3-L1 cells was not significantly decreased after exposure to either 200 or 150 μg/mL of OIE for 2 and 10 days, respectively compared to control cells. The OIE exhibited a dose-dependent reduction of lipid accumulation compared to the control (*p* < 0.05). The extract also demonstrated a dose-dependent inhibitory effect upon lipase activity compared to the control. The inhibitory effect of the OIE on lipid accumulation in 3T3-L1 cells was also confirmed using FTIR microspectroscopy. The signal intensity and the integrated areas relating to lipids, lipid esters, nucleic acids, glycogen and carbohydrates of the OIE-treated 3T3-L1 adipocytes were significantly lower than the non-treated 3T3-L1 adipocytes (*p* < 0.05). Principal component analysis (PCA) indicated four distinct clusters for the FTIR spectra of 3T3-L1 adipocytes based on biomolecular changes (lipids, proteins, nucleic acids, and carbohydrates). This observation was confirmed using Unsupervised hierarchical cluster analysis (UHCA).

**Conclusions:**

These novel findings provide evidence that the OIE derived from the fruit pods of the plant is capable of inhibiting lipid and carbohydrate accumulation in adipocytes and also has the potential to inhibit an enzyme associated with fat absorption. The initial observations indicate that OIE may have important properties which in the future may be exploited for the management of the overweight or obese.

## Background

Obesity has become a major global health problem [1]. It is a major contributor to diseases such as diabetes, cardiovascular disease and hypertension [2]. It has been established that being overweight or obese is associated with an increase in both the size and number of adipocytes along with an excessive amount of fat accumulation [3, 4]. Although using appetite suppressant drugs is a common way to treat obesity, long-term pharmacological treatment has been reported to generate many side-effects [5, 6]. This has led to innovative research using natural products to combat obesity in the hope of a safe and novel treatment [7, 8].

*O. indicum* belongs to Bignoniaceae family, widely found in Tropical Asia including Thailand. The chemical composition of *O. indicum* includes baicalein, chrysin, oroxylin A and oroxylin B [9, 10]. Many previous studies have reported antioxidant [11], anti-inflammatory [12], anti-diabetic [13] and hepatoprotective properties for *O. indicum* and its isolated compounds [14]. Animal studies suggest that *O. indicum* has few toxic effects and oral doses of 250 mg/kg BW for 28 days were tolerated well in rats [13]. It has previously been demonstrated that an extract of *O. indicum* derived from the root of the plant reduced the plasma concentrations of glucose, triglycerides and total cholesterol in diabetic rats [15]. Furthermore, stem bark extracts generated an inhibitory effect on an α-glucosidase enzyme in mature 3T3-L1 adipocytes [13]. Whilst, Oroxylin A, an isolated compound from *O. indicum,* induced both anti-adipogenesis and lipolysis in 3T3-L1 adipocytes. This was largely explained by a down-regulation of many transcriptional factors and an enhanced expression of pro-apoptotic proteins [16].

OIE has varying pharmacological properties depending on which part of the plant it is derived. In this study, we have used an extract from the fruit pods, rather than the stem of the plant which has been previously studied by several groups. The aim of this study was to provide a simple chemical composition of the extract. Initial experiments were performed using 3T3-L1 cells to establish any potential toxic effects of OIE and its impact on lipid accumulation within adipocytes. A further aim was to employ FTIR to examine the biochemical changes observed in the differentiated cells. An in vitro study was also conducted to examine the effect of OIE on pancreatic lipase. The results indicate that OIE can inhibit the development of adipocytes. Both treated and non-treated differentiated adipocytes had distinct biochemical profiles.

## Methods

### Chemicals

3T3-L1 mouse embryonic fibroblasts and bovine calf serum were purchased from the American Type Culture Collection (ATCC, CL-173, USA). Insulin solution from bovine, methyl isobutyl xanthine (IBMX), lipase from porcine pancreas type 2, dimethyl sulphoxide (DMSO), 4-Nitrophenyl dodecanoate (pNP), simvastatin and orlistat were purchased from Sigma-Aldrich (St. Louis, USA). Dulbecco’s modified Eagle’s medium with high glucose (DMEM), penicillin, streptomycin, N-2-hydroxyethylpiperazine-N-2-ethane sulfonic acid (HEPES) and 3-(4,5-Dimethylthiazol-2-yl)-2,5-diphenyltetrazolium bromide (MTT) were purchased from GIBCO Invitrogen (Grand Island, NY). Dexamethasone was acquired from G Bioscience (St. Louis, USA). Oil Red O was purchased from amresco (USA). Fetal bovine serum (FBS) was purchased from Hyclone (Logan, Utah).

### Preparation of *O. indicum* extract

*O. indicum* (fruit pods) fresh samples were purchased from the local market at Wang Nam Khiao district, Nakhon Ratchasima province, Thailand. The voucher specimens (SOI0808U) were deposited at the flora of Suranaree University of Technology (SUT) Herbarium and authenticated by Dr.Santi Wattatana, a lecturer and a plant biologist at Institute of Science, SUT, Thailand. Fresh pods were washed thoroughly with tap water, cut into small pieces and then dried in the oven at 40 °C for 2 days. The dried pieces were pulverised using a mechanical grinder, and the resulting coarse powder was preserved from moisture. The *O. indicum* dry powder (500 g) was extracted with 95% ethanol by a soxhlation for 8 h. The extract was filtered through Whatman filter paper and concentrated using a rotary evaporator at 50 °C under vacuum to remove the ethanol. The remaining extract was stored at − 80 °C until required. Subsequently, the sample was lyophilized in a freeze dryer (LABCONCO), automatic mode, vacuum 240 × 10^− 3^ mBar, and collector − 55 °C. The extracted powder was stored at − 20 °C until required. The lyophilized *O. indicum* extract was used within 3 months of preparation. The extract was resuspended in the differentiation medium containing 0.1 *v*/v DMSO (vehicle) and added to the cells at concentrations ranging from 0 to 1500 μg/mL.

### Phytochemical screening

The stock concentration of the lyophilized extract (10 mg/mL) was prepared and tested for the presence of bioactive phytochemical compounds, including total phenolics, flavonoids, alkaloids, steroids, glycosides, tannin, and saponins.

### Test for flavonoids

Briefly, 1 mL of the OIE was mixed with 2 mL of 2% *w*/*v* of NaOH. A 2 mL aliquot of 10% w/v lead acetate solution was added to 1 mL of the alkaline extract. The formation of a yellow colour indicated the presence of flavonoids [17, 18].

### Test for alkaloids

The Mayer’s and Wagner’s test was performed. Briefly, 1 mL of the OIE was added to 2 mL of 1% *v*/v HCl and heated. A few drops of Mayer’s and Wagner’s reagents was added to the mixture, the presence of a precipitate suggested the presence of alkaloids in the sample [17, 18].

### Test for steroids

The steroids containing in OIE were investigated. In short, 1 mL of the OIE was mixed with 2 mL each of chloroform and concentrated H_2_SO_4._ A red colour in the lower layer indicated the presence of steroids [17, 18].

### Test for glycoside

The detection of glycosides was performed using Salkowski’s test. In brief, 2 mg of the OIE was mixed with 2 mL of chloroform and a few drops of concentrated H_2_SO_4_. A reddish brown colour was associated with the presence of glycosides [17, 18].

### Test for tannin

The Gelatin test was performed to identify tannins in the extract. To summarise, 1 mL of the OIE was added to 2 mL of a 1% *w*/*v* gelatin solution, the formation of a white precipitate indicated the presence of tannins [17, 18].

### Test for saponin

The foam test was performed. Briefly, a 5 mL aliquot of the OIE was shaken for 5 min. If saponins were present, they formed a stable foam in the test tube [17, 18].

### Total phenolic content determination (TPC)

The total phenolic content was measured following the method outlined by Kohoude with slight modifications [19]. In brief, 20 μL of extract (0.625 mg/mL) or a standard solution of gallic acid (0–7.5 μg/mL) were added into 100 μL of Folin-Ciocalteu reagent. The mixture was incubated at room temperature for 6 min, prior to the addition of 7.5% *w*/*v* of Na_2_CO_3_. After a 1 h incubation at room temperature, the absorbance was recorded at 760 nm against a DMSO blank. TPC of the sample was expressed as mg of gallic acid equivalents (GAE) per g of dry weight**.**

### Total flavonoid content determination (TFC)

The total flavonoid content was measured according to a previously published method with slight modifications [20]. To summarise, a 25 μL of extract (3 mg/mL) or a standard solution of catechin (0–200 μg/mL) were added to 125 μL of deionised water, followed by the addition of 10 μL of 5% *w*/*v* NaNO_2_. This mixture was incubated at room temperature, and after 6 min, 15 μL of 10% AlCl_3_ was added. Upon mixing the reaction was allowed to proceed for 5 min, then 50 μL of 1 M NaOH was added. The absorbance of the mixture was determined at 595 nm versus prepared DMSO blank. Total flavonoid of the sample was expressed as mg of catechin equivalent (CE) per g of dry weight.

### Cell culture and differentiation procedures

The differentiation procedures of 3T3-L1 preadipocyte were performed as following the ATCC recommended protocol. Briefly, adherent 3T3-L1 cells were cultured in DMEM containing a high glucose concentration, supplemented with 10% of bovine calf serum, 100 U/mL of penicillin and 100 μg/mL of streptomycin, until they reached 70–80% confluently. Two days after confluence (day 0), the cells were stimulated to differentiate with differentiation medium containing 10% FBS, 1.0 μM dexamethasone, 0.5 mM of IBMX, and 1.0 μg/mL of insulin in DMEM. On day 2, the differentiation medium was changed to maintain a medium consisting of 10% of FBS and 1.0 μg/mL of insulin in DMEM. The maintenance medium was replaced every 48 h for the next 8 days. On day 10, the differentiation of 3T3-L1 pre-adipocytes into adipocytes was observed. The cells were maintained in 5% CO_2_ incubator and at 37 °C throughout the whole process**.** The required doses of OIE were added to the 3T3-L1 cell culture during the differentiation (at day 0, 2, 4, 6, and day 8).

### Cytotoxicity assay

The cytotoxic effects of the OIE on the proliferation of preadipocytes and adipocytes were determined by MTT assay largely following the method of Dunkhunthod et al. and Denizot [21, 22]. In brief, the 3T3-L1 cells were seeded in 96-well plates at a density of 5 × 10^3^ cells/well. Two days after reaching confluence, the dividing cells were treated with the OIE at concentrations ranging from 0 to 1500 μg/mL. Both treated and control cells were incubated for a further 48 h. At the end of the treatment period, the cell viability was assessed by using the MTT assay. The culture medium was removed, and 100 μL of MTT solution (0.5 mg/mL in phosphate buffer saline) was added, then incubated at 37 °C for 4 h. After incubation, 150 μL of DMSO was added to dissolve formazan crystal. The absorbance of the intracellular formazan is proportional to the number of viable cells present was determined at 540 nm against a blank medium (Benchmark Plus, Bio-Rad, Japan). The percentage of formazan product was calculated to determine cytotoxicity [23]. OIE at concentrations of 0–200 μg/mL were used to assess any cytotoxic effects of mature adipocytes and Oil Red O assay for lipid accumulation. The IC_50_ of the extract was also calculated from a dose-response curve using linear regression analysis.

### Oil red O staining

The intracellular triglyceride content was determined using an Oil Red O staining method as previously described [21, 24]. Briefly, on day 10, cells were washed with PBS and fixed with 1 mL/well of 10% (*v*/v) formalin for 1 h at room temperature. After fixation, the cells were washed, and 500 μL of 0.5% of the Oil Red O solution was added. The cells were incubated for 30 min at room temperature. The Oil Red O solution was removed by gentle aspiration, and the cells were washed with PBS. The nucleus was then stained with 0.10% (*w*/*v*) haematoxylin. Fat droplets were observed under an inverted microscope at an appropriate magnification. To determine the percent of lipid accumulation, the cells were extracted with 250 μL of isopropanol and 200 μL of the eluted solution was transferred to a new 96 well plate. The absorbance was measured at 490 nm with a microplate spectrophotometer. The simvastatin at 1.67 μg/mL was used as a positive control. The 3T3-L1 cells, treated with 200 μg/mL OIE were selected for FTIR studies.

### Fourier-transform infrared spectroscopy

The effect of OIE on 3T3-L1 adipocyte cells using FTIR measurement was performed following the method of Dunkhunthod et al. [21]. Briefly, on day 10, cells were collected and centrifuged at 4000×*g* for 5 min, the medium was removed by gentle aspiration, and the cells were agitated and washed with 0.85% *w*/*v* NaCl. The cell suspensions were centrifuged at 4000×*g* for 5 min. The acquired cell pellets were dropped onto a window slide (MirrIR, Kevley Technologies) and dried for 30 min in a desiccator to eliminate the excess water. The dried cells were stored in a desiccator prior to FTIR analysis.

FTIR spectra were obtained at the Synchrotron Light Research Institute (Public Organization), Thailand. FTIR spectra were acquired with a Bruker Vertex 70 spectrometer coupled with a Bruker Hyperion 2000 microscope (Bruker Optics Inc., Ettlin-Gen, Germany) equipped with nitrogen cooled MCT (HgCdTe) detector with a 36 x IR. The spectra were obtained in the reflection mode with the wavenumber range of 4000–600 cm^− 1^, using an aperture size of 50 μm × 50 μm, with a resolution of 6 cm^− 1^. Each spectrum was produced following 64 scans. OPUS 7.2 software (Bruker Optics Ltd., Ettlingen, Germany) was used to acquire FTIR spectral data and control instrument system.

The spectral ranges of biochemical interest were identified using Principal Component Analysis (PCA) as being between 3000 and 2800 cm^− 1^ and 1800–850 cm^− 1^. The preprocessing of the spectra was performed by second derivative transformations using the Savitzky-Golay algorithm (nine smoothing points) and normalised with extended multiplicative signal correction (EMSC). Score plots (3D) and loading plots were used to represent the different classes of data and relations among variables of the data set, respectively.

The FTIR spectra datasets were submitted for Unsupervised Hierarchical Cluster Analysis (UHCA), to collect similar spectra in groups or clusters, using the OPUS 7.2 software (Bruker). Cluster analysis was performed on the second derivatives, and vector normalises spectra using Ward’s algorithm.

### Lipase activity

Measurement of lipase activity was performed as previously described by Guo et al. and Dunkhunthod et al. [21, 25]. In brief, lipase of porcine pancreas type 2 was dissolved in distilled water at 5 mg/mL, the solution was centrifuged at 10,000 *xg* for 5 min, and the supernatant was used for the assay. A 0.1% *w*/*v* solution of pNP laurate was prepared in 5 mM of sodium acetate (pH 5.0) containing 1% *v*/v Triton X-100. The solution was heated to 80 °C and cooled to room temperature prior to use. A 30 μL volume of the lipase was added to a 96 well plate, followed by 40 μL of reaction buffer (100 mM of Tris buffer pH 8.2). Either 20 μL of OIE or 50% v/v DMSO was added prior to the addition 30 μL of the substrate solution. The mixtures were incubated at 37 °C for 6 h and measured at 409 nm using a microplate spectrophotometer. Orlistat at 12.5 to 100 μg/mL was used as a positive control. The inhibition rate (%) was calculated using the following equation. [((OD _control_ – blank _control_) – (OD _sample_ – blank _sample_)) /OD _control_] × 100 [26].

### Statistical analysis

All the data were expressed as a mean ± standard error of the mean (SEM). The statistical significances difference between treatment and control groups of cell viability, the amount of lipid accumulation, biomolecular changes, and lipase activity were analysed by One-way analysis of variance (ANOVA) with a Turkey’s HSD post-hoc test (SPSS v 23). Values were considered statistically significant when *p* < 0.05 and data were representative of at least three independent experiments (*n* ≥ 3). Most experiments were performed in triplicate.

## Results

### The phytochemical composition, TPC, and TFC

A 1.0 Kg weight of fruit pods from *O. indicum* was processed to obtain a final yield of 18.41% (*w*/w) OIE (Table 1). Qualitative tests revealed that the extract contained flavonoids, alkaloids, steroids, glycoside, and tannins, but no saponins were detected. The concentration of TPC and TFC present in OIE were 51.483 ± 0.766 mg GAE/g and 34.191 ± 2.473 mg CE/g of dry weight, respectively (Table 1).

**Table 1:** Extraction yield and phytochemical composition of *Oroxylum indicum* fruit extract. Extraction yield, total phenolic content, total flavonoid content and phytochemical composition of *Oroxylum indicum* fruit extract

Sample	Fresh weight (g)	Dry weight (g)	Yield (g)	Yield (%w/w)	Flavonoids	Alkaloids	Steroids	Glycoside	Tannins	Saponin
*Oroxylum indicum*	1000	133.40	24.53	18.41	+	+	+	+	+	–
*Total phenolic content (TPC)*		51.48 ± 0.77 (mg GAE/g)^a^								
*Total flavonoids content* *(TFC)*		34.19 ± 2.47 (mg CE/g)^a^								

(**+) =** positive test; (−) = negative test

Weight and yield of *Oroxylum indicum* was calculated from a 1.0 kg extraction of raw material. ^**a**^The TPC and TFC were derived from calibration curves using the relevant standard and based on dry weight of *Oroxylum indicum***.** The data are expressed as the mean ± SEM of three independent experiments performed in triplicate (*n* = 9)

### The effect of OIE on the viability of pre- and mature adipocytes

**Fig. 1:**
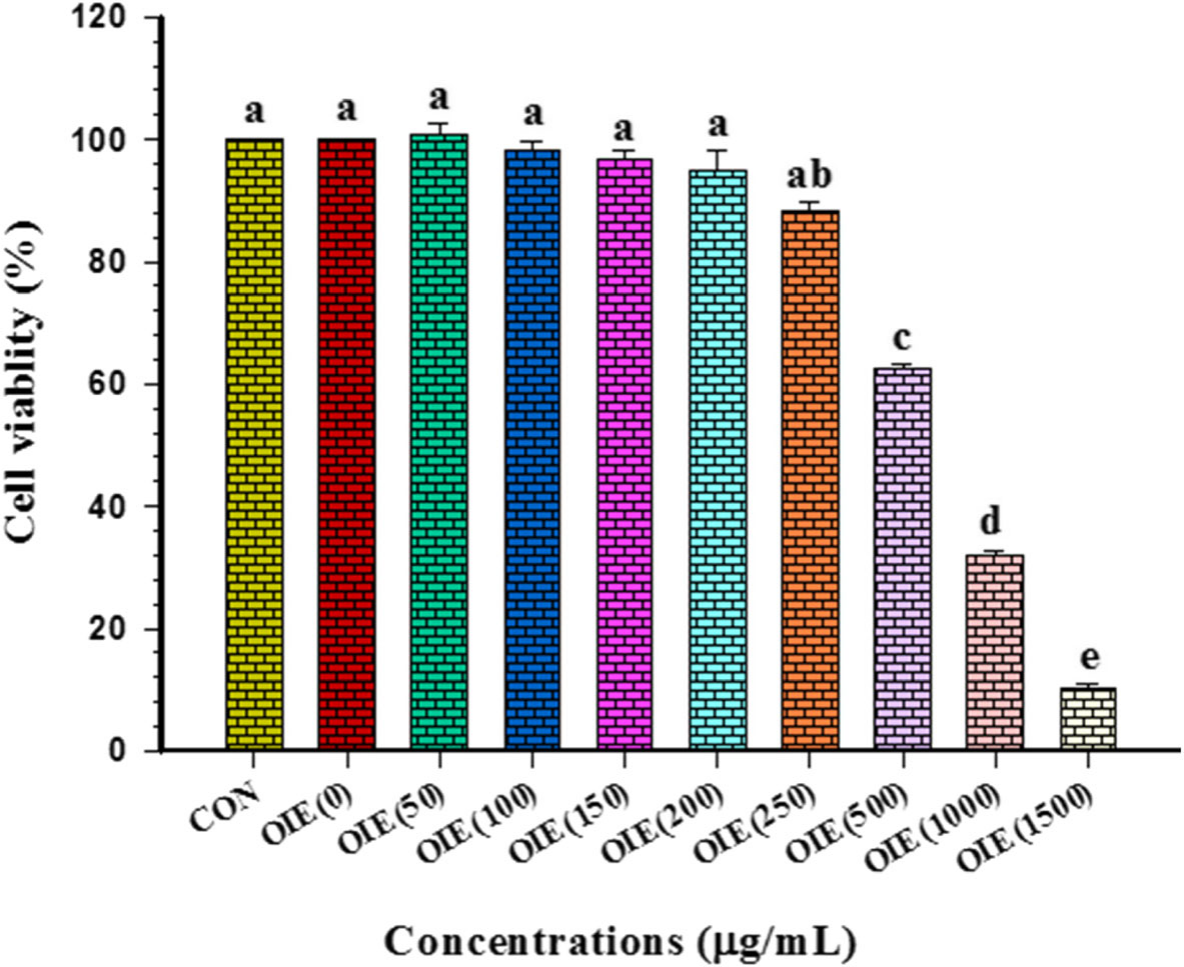
Graphically represents the effect of *Oroxylum indicum* extract on the viability of 3T3-L1 preadipocytes. CON = vehicle control; OIE(50) = *Oroxylum indicum* at 50 μg/mL. Values are Means ± SEM (*n* = 3 replicates). A significant differences were observed (Tukey’s HSD test, *p* < 0.05) and are represented on the figure with letters from a to e. Bars annotated with the differrent letters are significantly different to the control

To evaluate the cytotoxicity of OIE on both non-differentiated and differentiated 3T3-L1 cells, an MTT assay was conducted. A concentration of OIE between 250 to 1500 μg/mL showed a significant reduction (*p* < 0.05) in the viability of pre-adipocytes (Fig. 1). However, the viability of cells treated with lower doses of the extract (50 to 200 μg/mL) was not significantly different compared to non-treated cells (*p* > 0.05). The IC_50_ at 48 h for pre-adipocytes was 882.68 ± 47.99 μg/mL. These results suggest that doses of the OIE above 250 μg/mL are detrimental to pre-adipocytes viability. In the light of this observation, all subsequent experiments to investigate mature adipocyte viability used a dose of 200 μg/mL or less.

**Fig. 2:**
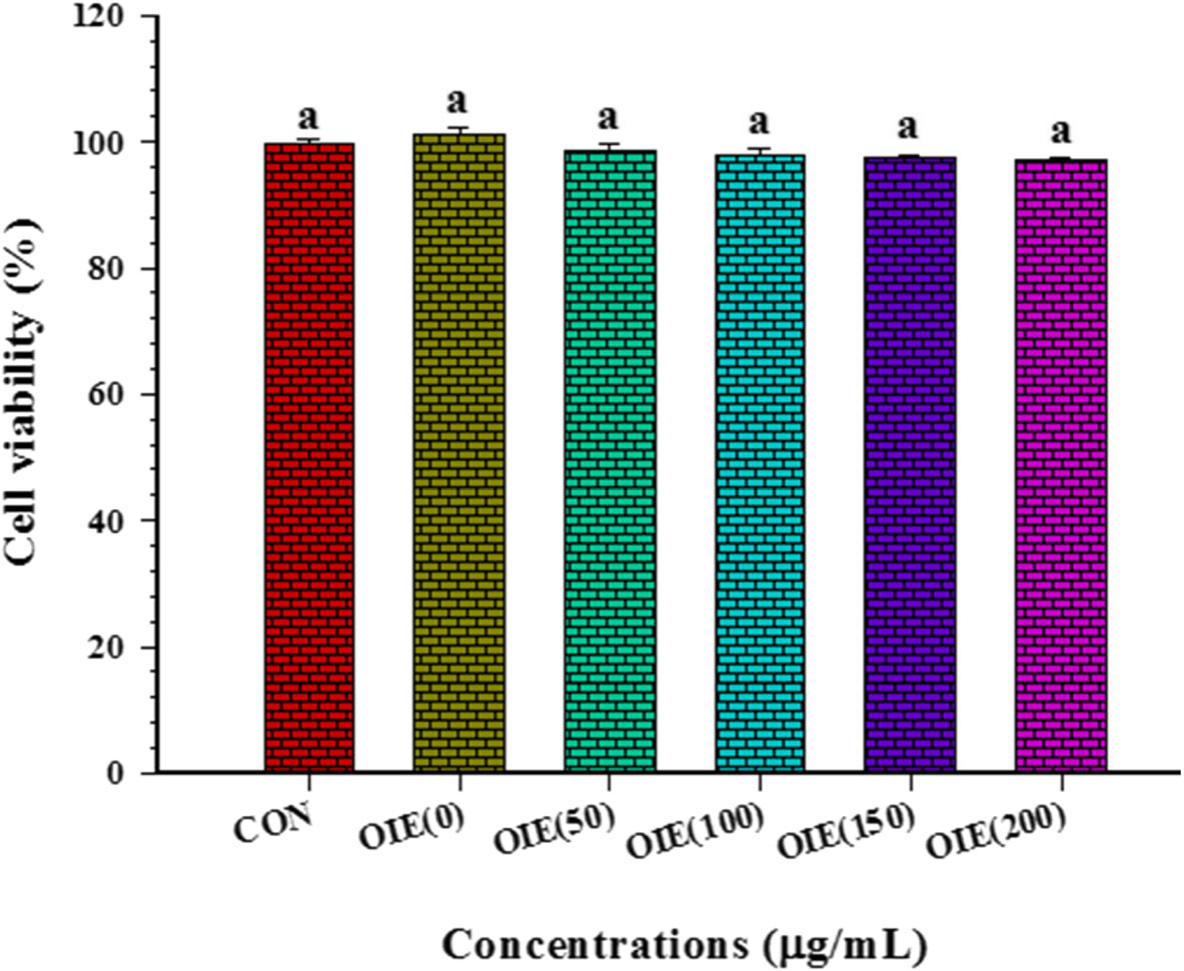
Graphically represents the effect of *Oroxylum indicum* extract on the viability of 3T3-L1 mature adipocytes. CON = vehicle control; OIE(50) = *Oroxylum indicum* at 50 μg/mL. Values are Means ± SEM (*n* = 3 replicates). For all treatments no significant differences were observed (Tukey’s HSD test, *p* < 0.05)

The effect of the OIE on mature adipocytes indicated that doses of OIE at 50 to 200 μg/mL did not reduce the viability of mature cells compared to the control (*p* > 0.05) over a 10 day period (Fig. 2).

### The effect of OIE on adipocyte differentiation and lipid accumulation

**Fig. 3:**
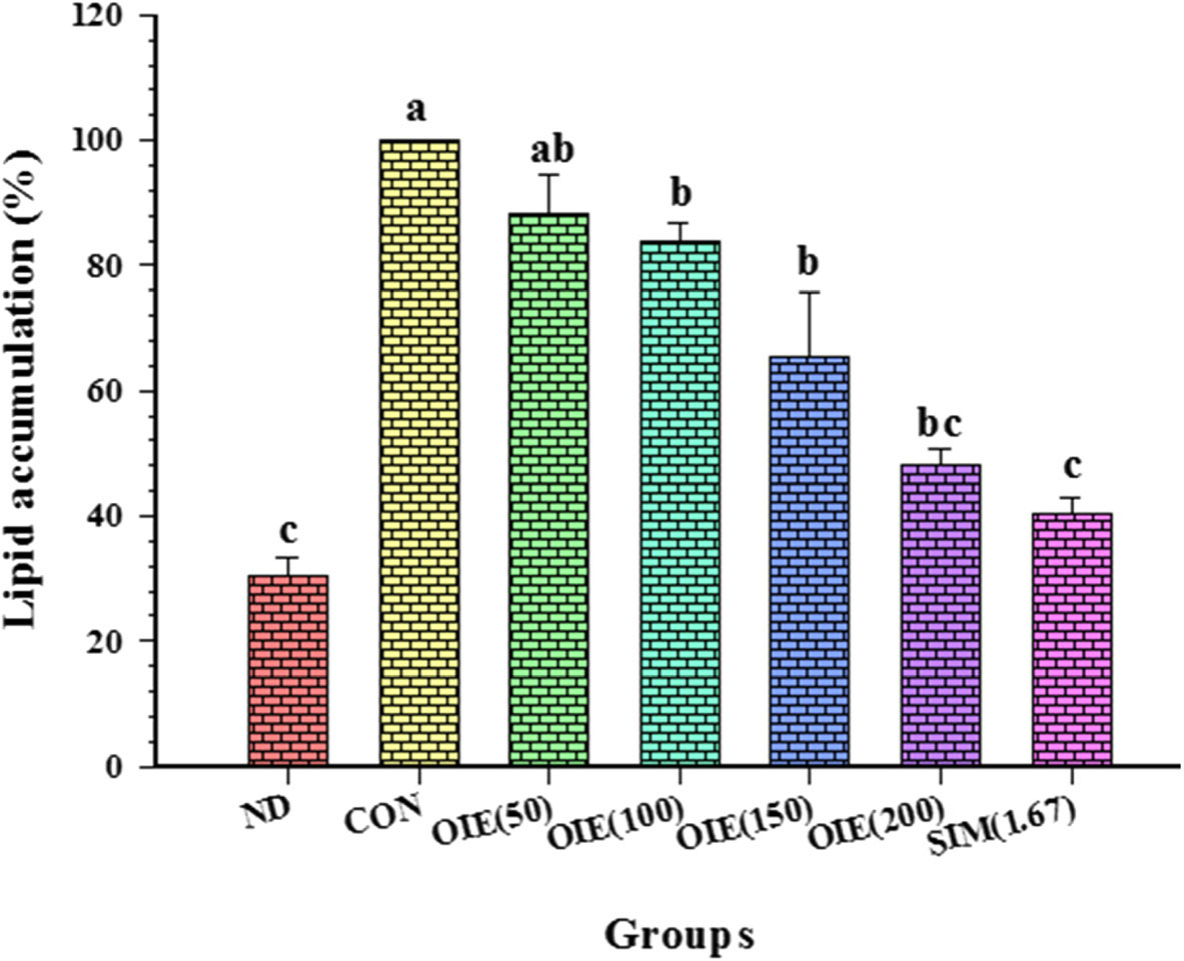
The effect of *Oroxylum indicum* extract on the accumulation of triglycerides in 3T3-L1 differentiated cells. ND = non-differentiated cells (pre-adipocytes); CON = vehicle control; OIE(50) = *Oroxylum indicum* at 50 μg/mL. SIM(1.67) = Simvastatin at 1.67 μg/mL (a positive control). Values are Means ± SEM (*n* = 3 replicates). A significant differences were observed (Tukey’s HSD test, *p* < 0.05) and are represented on the figure with letters from a to c

During the differentiation of 3T3-L1 preadipocytes to adipocytes, the cells were treated with OIE, and the intracellular lipid concentration was determined using Oil Red O staining. The 3T3-L1 pre-adipocytes when exposed to the differentiation medium resulted in a significant increase of lipid accumulation in comparison to pre-adipocytes (*p* < 0.05) (Fig. 3). However, the addition of OIE at 200 μg/mL significantly decreased the intracellular lipid accumulation by approximately 52%, compared to the 3T3-L1 adipocyte (control) (*p* < 0.05). The IC_50_ and IC_60_ values for OIE on lipid accumulation were determined to be 201.26 ± 10.00 and 237.72 ± 14.96 μg/mL, respectively. In addition, the cholesterol-lowering drug, simvastatin exhibited an IC_60_ at a concentration of 1.67 μg/mL. The effect of simvastatin is 142 times greater than OIE.

**Fig. 4:**
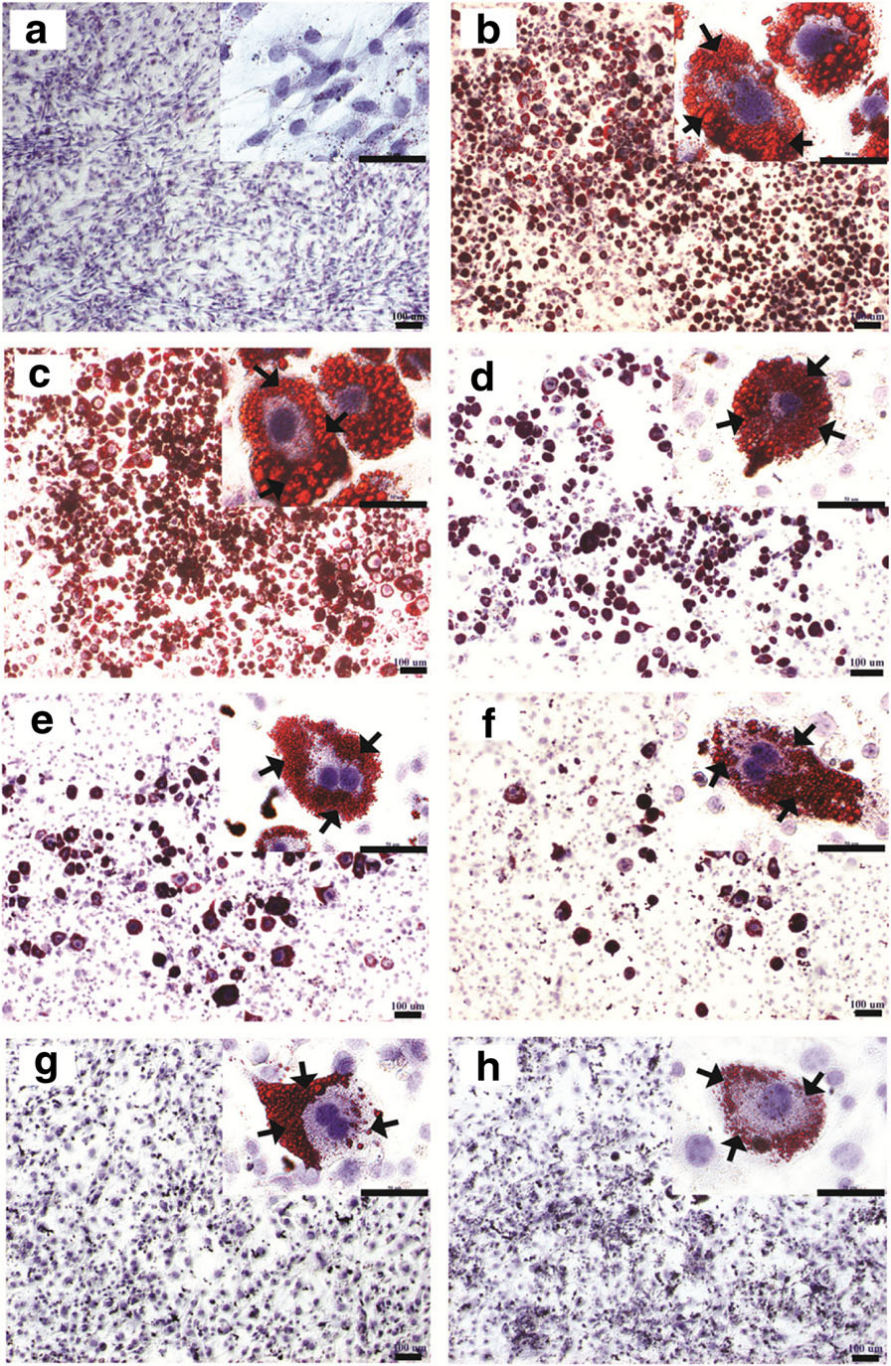
Microscopic imaging of intracellular lipid after Oil Red O and heamatoxylin staining of the samples. 3T3-L1 cells are stained with Oil Red O and heamatoxilin. The lipid droplets were red in appearance and the nuclei were blue in colour. The arrows indicate lipid droplets within the adipocytes. The size and the number of lipid droplets appear to be a larger in untreated adipocytes (**b** and **c**). **a** non-differentiated cells (pre-adipocytes); **b** differentiated cells (untreated adipocytes); **c** vehicle control; **d**, **e**, **f** and **g** differentiated cells treated with *Oroxylum indicum* extract at the dose 50 μg/mL, 100 μg/mL, 150 and 200 μg/mL, respectively; **h** simvastatin at 1.67 μg/mL (original magnification at ×40, scale bar; 100 μm and *Inset* view at × 400, scale bar; 50 μm)

In order to gain more evidence that OIE could affect lipid accumulation in differentiated 3T3-L1 cells, pre-adipocytes and adipocytes were stained with Oil Red O and heamatoxylin and observed a light microscope (Fig. 4). Untreated adipocytes displayed an increase in size and number of prominent lipid droplets (Fig. 4b). Whilst adipocytes treated with simvastatin exhibited very small and diffuse lipid droplets (Fig. 4h). The 200 μg/mL OIE treated adipocytes displayed smaller size and number of lipid droplets that seemed to be focused on one part of the cell (Fig. 4g).

### FTIR spectra profiles

**Fig. 5:**
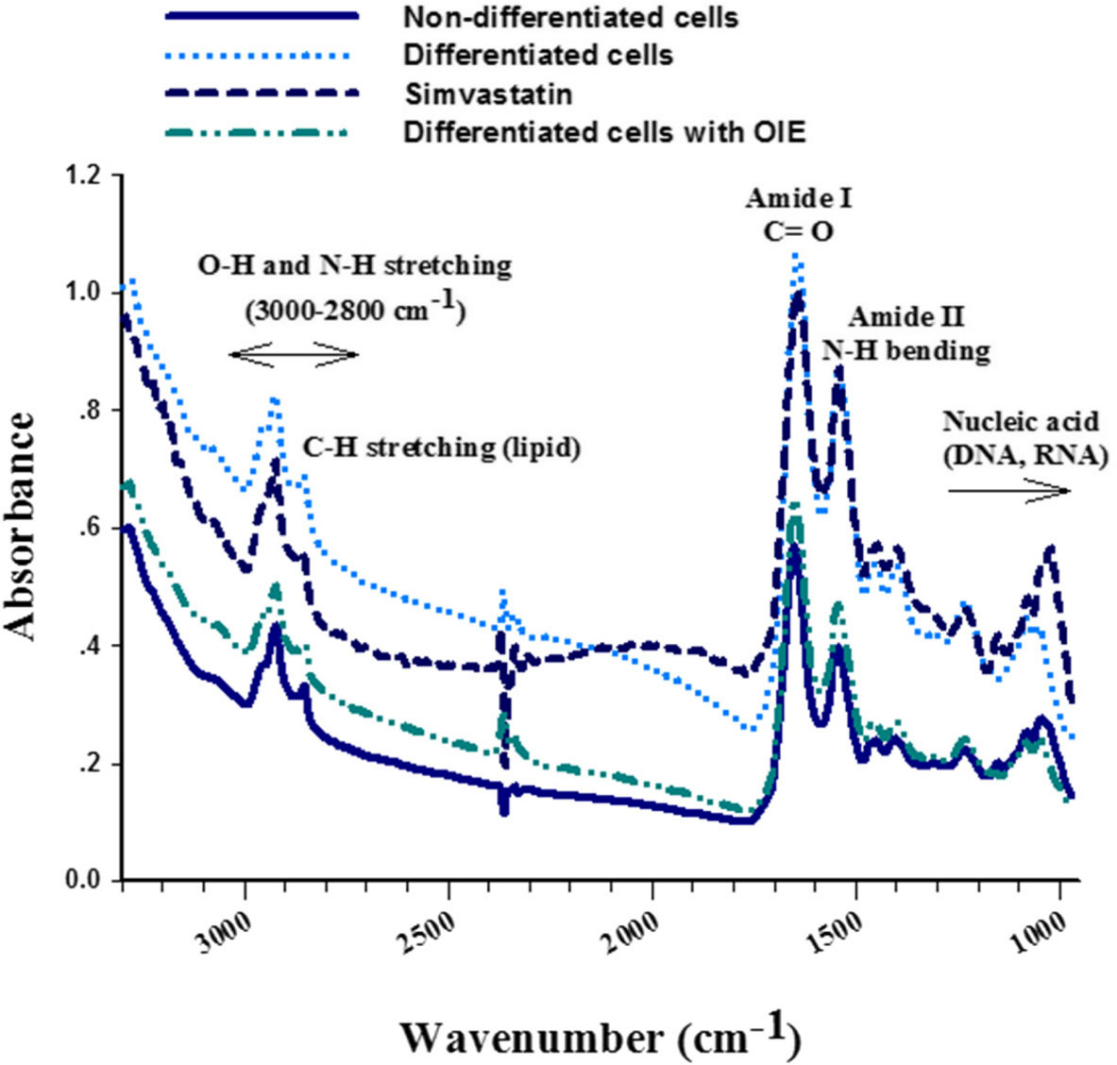
Average original FTIR spectra (3000–950 cm^− 1^) obtained from 3T3-L1 cells. The raw spectra of non-differentiated cells (preadipopcytes) (*n* = 62), differentiated cells (*n* = 92), simvastatin and *Oroxylum indicum* extract treated 3T3-L1 adipocytes (*n* = 47) after 10 days

FTIR microspectroscopy was used to determine the biochemical composition of pre-adipocytes, adipocytes, and adipocytes treated with either, simvastatin or OIE. For each treatment FTIR spectra between wavelengths 3000–950 cm^− 1^ were obtained (Fig. 5). The Spectra were converted to second order derivatives to allow a more detailed comparison of the different treatments to the cells (Table 2). The spectra were separated into three distinct areas: (1) lipids (3000–2800 cm^− 1^), (2) proteins (1700–1500 cm^− 1^) and (3) carbohydrate and nucleic acids (1300–950 cm^− 1^).

**Table 2:** FTIR band assignments for functional groups found in the second derivative spectra of 3T3-L1 cells [27, 28]

2nd derivative spectra (cm^− 1^)	Band assignment
2962	Asymmetrical stretching (C-H) from methyl (-CH_3_) groups of lipids
2924	Asymmetrical stretching (C-H) from methylene (-CH_2_) groups of lipids
2854	Symmetrical stretching (C-H) from methylene (-CH_2_) groups of lipids
1740	(C=O) of ester functional groups primarily from lipid and fatty acids
1647	Amide I protein (C=O stretching)
1543	Amide II protein (N-H bending, C-H stretching)
1462	Asymmetrical deformation (CH_2_)_scissor_ from methylene (-CH_2_) groups of lipids
1400	Symmetrical stretching (COO^−^) associated with symmetrical in-plane deformation bend (CH_3_) of proteins
1234	Asymmetrical stretching (PO_2_^−^) mainly nucleic acids with the little contribution from phospholipids
1153	Asymmetrical stretching (CO-O-C) of glycogen, other carbohydrates and nucleic acids
1080	Symmetrical stretching (PO_2_^−^) of the phosphodiester backbone of nucleic acids (DNA and RNA) and phospholipids
1018	(C-O) vibration from glycogen and other carbohydrates

### The second derivative spectra in the lipid region

**Fig. 6:**
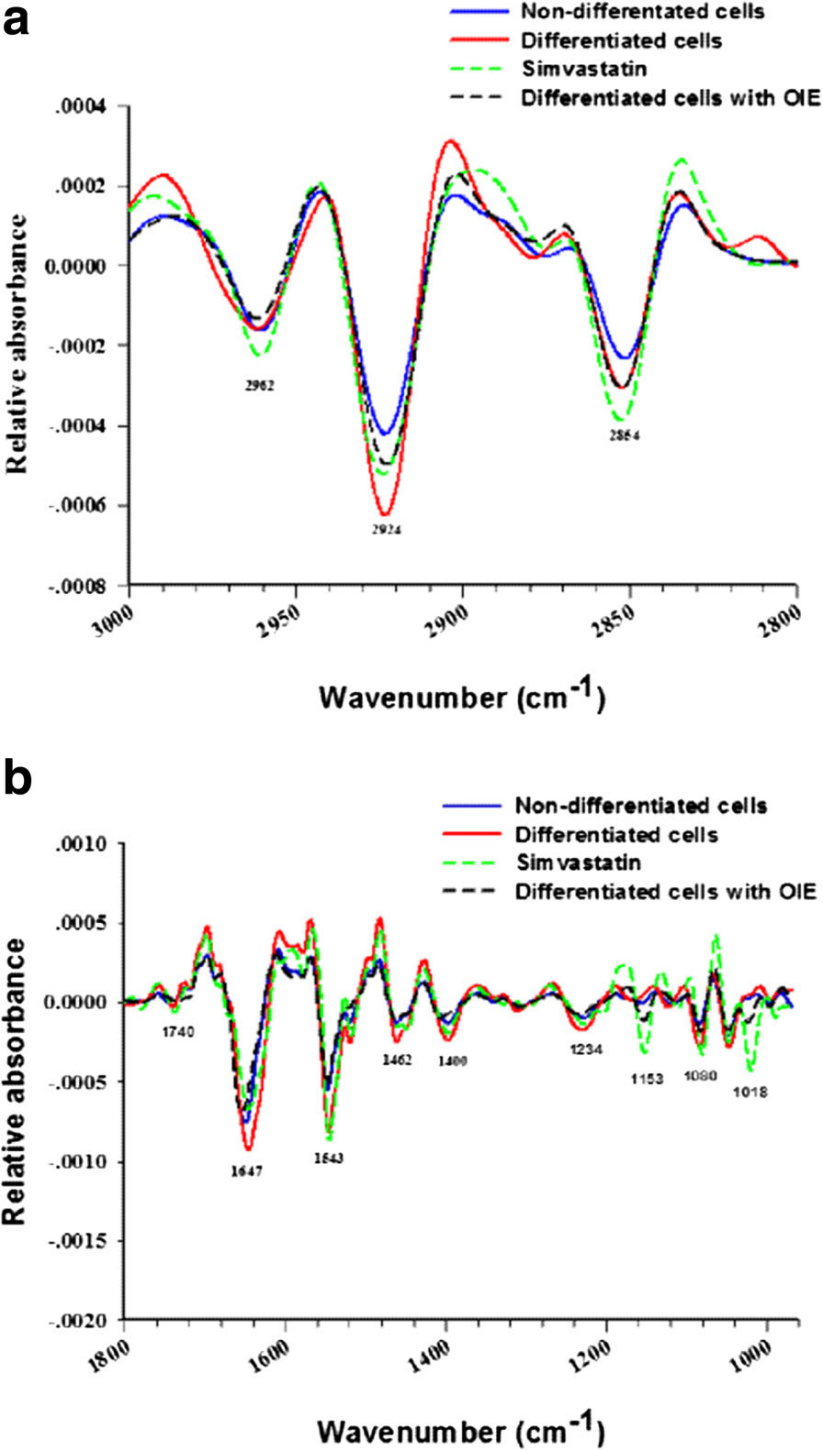
Average the secondary derivative spectra of 3T3-L1 cells. The second derivative spectra of non-differentiated cells (pre-adipocytes), differentiated cells, simvastatin and *Oroxylum indicum* extract–treated differentiated cells after 10 days. The data were represented in two regions: **a** lipid regions (3000–2800 cm^− 1^) and **b** protein, nucleic acid, glycogen and other carbohydrate regions (1800–950 cm^− 1^)

**Fig. 7:**
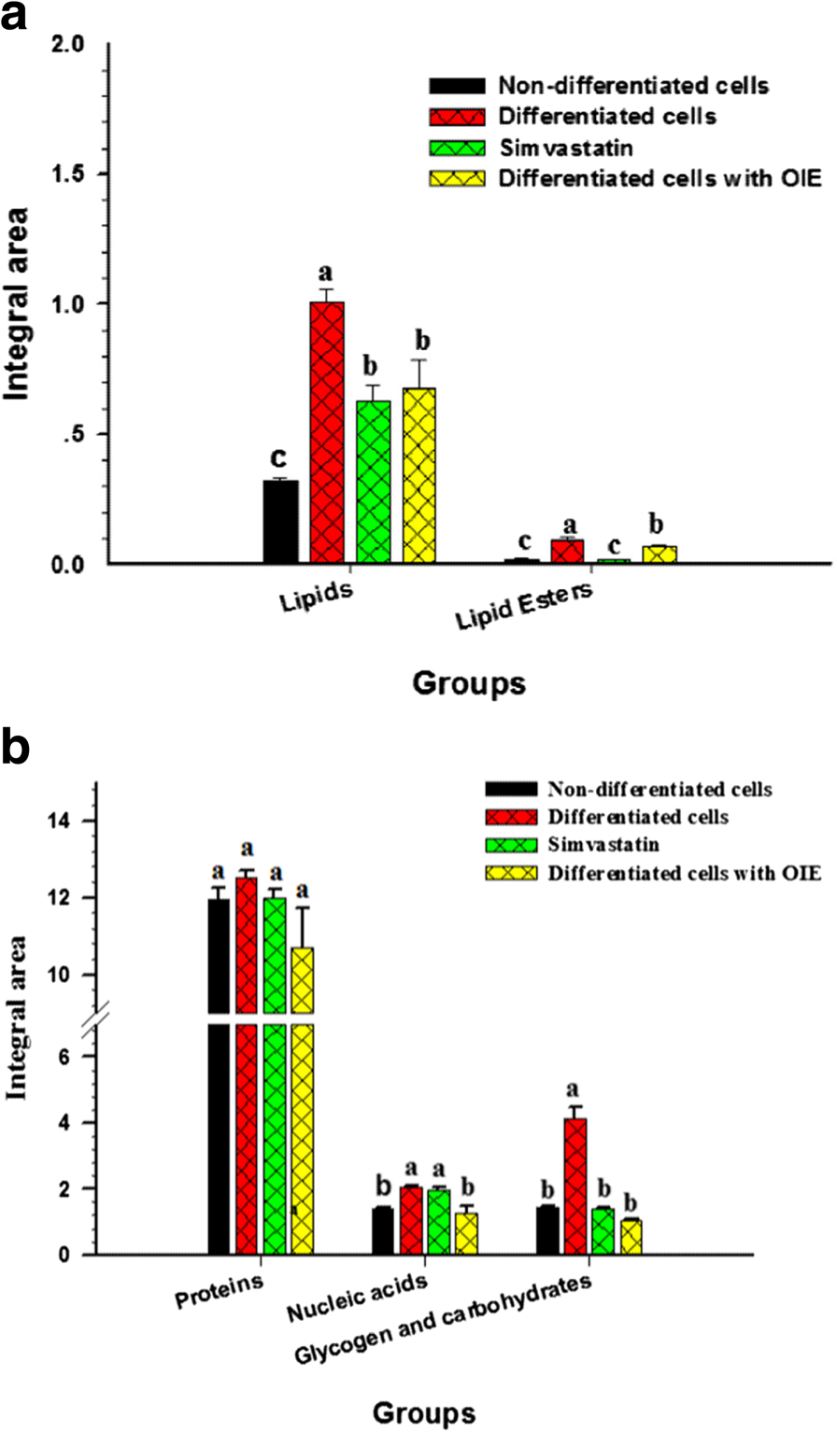
The histogram of integrated areas of 3T3-L1 cells. The integrated area from second derivative data of non-differentiated cells (pre-adipocytes), differentiated cells, simvastatin- and *Oroxylum indicum* extract-treated differentiated cells. Integral area of lipids and lipid esters (**a**). Integral area of proteins, nucleic acids, glycogen and carbohydrates (**b**). The results reported as means ± SEM (*n* = 3). Significant differences were observed (Tukey’s HSD test, *p* < 0.05) and are represented on the figure with letters from a to c

The spectra in the high-frequency region at 3000–2800 cm^− 1^ correspond to the symmetrical and asymmetrical vibrations of –CH groups of the lipid content [27]. The average second derivative spectra of 3T3-L1 cells under different experimental conditions exhibited three characteristic regions at 2962 cm^− 1^, 2924 cm^− 1^ and 2854 cm^− 1^ which are associated with cellular lipids. Each area is produced as a result of asymmetrical stretching from the methyl and methylene groups of lipids, and also the symmetrical stretching from methylene groups of lipids (Fig. 6a). Further evidence for the presence of lipids occurred at the lower wavenumber region at 1740 cm^− 1^, which is assigned to C=O stretches of the ester functional groups from lipids and fatty acids (Fig. 6b) [28]. The relative absorbance at 2924 cm^− 1^ and 1740 cm^− 1^ of adipocyte were stronger than the treated cells or pre-adipocytes (Fig. 6a and b). To discriminate between non treated and treated adipocytes the ratio of the integrated area of several functional groups was calculated. The selected regions were CH_2_ (2938–2906 cm^− 1^, centred at 2924 cm^− 1^)/CH_3_ (2973–2954 cm^− 1^, centred at 2962 cm^− 1^) [29, 30]. The selection of these area was made as they are associated with asymmetric stretching of lipids. The results showed that the ratio of integrated area of the lipids region in the OIE-treated adipocytes displayed significantly less than the untreated adipocytes group (*p* < 0.05) and did not significant from a simvastatin-treated group (*p* > 0.05) (Fig. 7a). Another useful parameter was the integrated area of the ester functional groups from lipids at 1740 cm^− 1^ in the OIE-treated adipocytes group indicated significantly lower than the untreated adipocytes group (*p* < 0.05) (Fig. 6b and 7a).

### The second derivative spectra in the protein region

Spectra in the areas from 1700 to 1500 cm^− 1^ are attributed to an absorption peak of proteins amide I and II. The ratio of the integrated area of several proteins related functional groups, including CH_2_ asymmetric stretching (2938–2906 cm^− 1^, centred at 2924 cm^− 1^)/amide I (1674–1624 cm^− 1^, centred at 1647 cm^− 1^) was calculated for control and treated adipocytes. In this case there no significant difference between non treated adipocytes and OIE treated adipocytes (*p* > 0.05) (Fig. 7b) [31].

### The second derivative spectra in the carbohydrate and nucleic acid region

C-O vibrations attributed to glycogen and other carbohydrates were identified at wavelengths of 1153 cm^− 1^ and 1018 cm^− 1^ [32]. These signal intensity and integral area of the OIE-treated adipocytes displayed significantly less than untreated adipocytes (*p* < 0.05) but not significant different from simvastatin-treated group (Fig. 7b).

The functional group of PO_2_ stretching mode from mainly nucleic acids was located at 1234 cm^− 1^ and 1080 cm^− 1^. In this case, the OIE-treated adipocytes displayed signal intensity and integral area significantly less than the untreated and simvastatin-treated adipocytes (*p* < 0.05) (Fig. 6b and 7b) [27].

### PCA

**Fig. 8:**
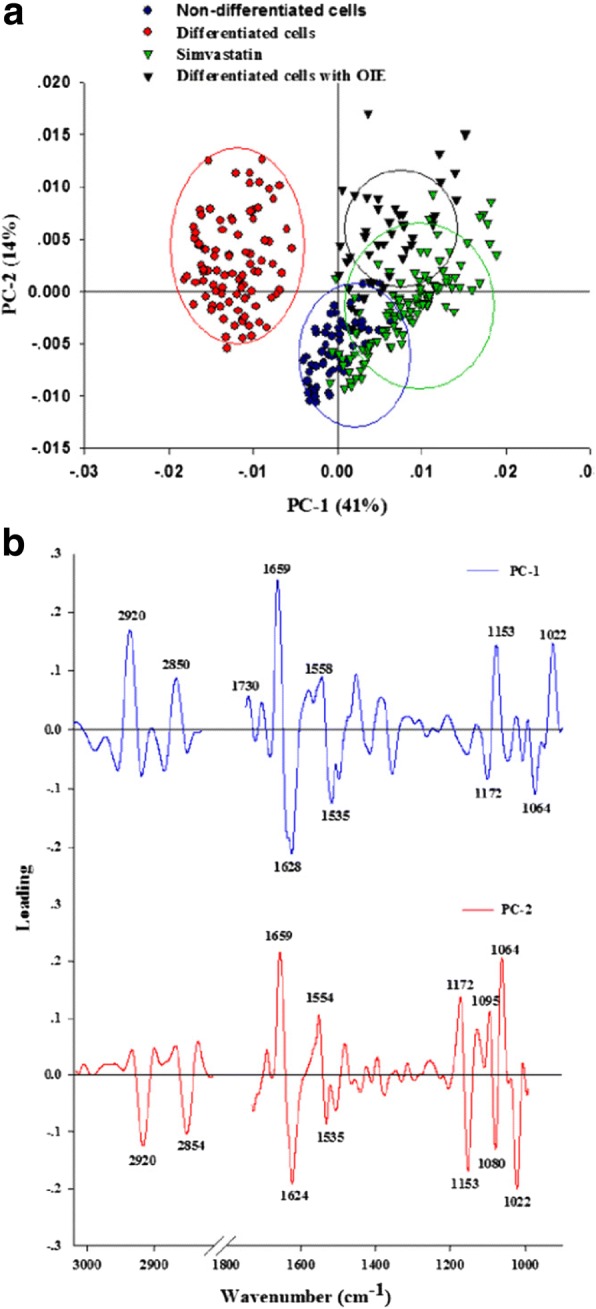
Principal component analysis (PCA) of FTIR spectral ranges 3000–2800 cm^− 1^ and 1800–950 cm^− 1^ giving PCA score plot. The 2D PCA score plots showed the clustering separation spectra between 3T3-L1 non-differentiated cells, differentiated cells, simvastatin- and *Oroxylum indicum* extract-treated differentiated cells after 10 days (**a**). PCA loading plot (**b**). The biomarker differences over a spectral range of samples are identified by the PC1 and PC2 loading plots

Principal Component Analysis (PCA) was performed to further discriminate data acquired from pre-adipocyte, untreated adipocyte, simvastatin- and OIE-treated adipocytes using FTIR. The PCA results were obtained from second-order derivative spectra at 3000–2800 cm^− 1^ and 1700–950 cm^− 1^. The results from 2-dimensional PCA clustering (Fig. 8a) clearly reveal district separation of differentiated adipocytes from treated adipocytes. The OIE and simvastatin-treated adipocytes would appear to have similar properties. Also, the spectrum band which most influences to the clustering can be examined through the PCA loading plots (Fig. 8b). PC1 loading plot was discriminated by the loading spectra at 2920 cm^− 1^ and 2850 cm^− 1^ caused by the C-H stretching, and at 1153 cm^− 1^ and 1022 cm^− 1^ resulting from the C-O vibrations from glycogen and other carbohydrate [33], which separated the negative score plot of the untreated adipocytes from the positive score plot of simvastatin- and OIE-treated adipocytes These results illustrated and confirmed that untreated adipocytes had a higher lipid and carbohydrate contents than simvastatin- and OIE-treated adipocytes. Moreover, the negative PC2 loading in the lipids region (centred at 2920 cm^− 1^ and 2854 cm^− 1^_,_ 1485 cm^− 1^) suggest that preadipocytes and OIE-treated adipocytes exhibit different properties. These results are consistent with the second derivative spectra results mentioned in the previous section.

### UHCA

**Fig. 9:**
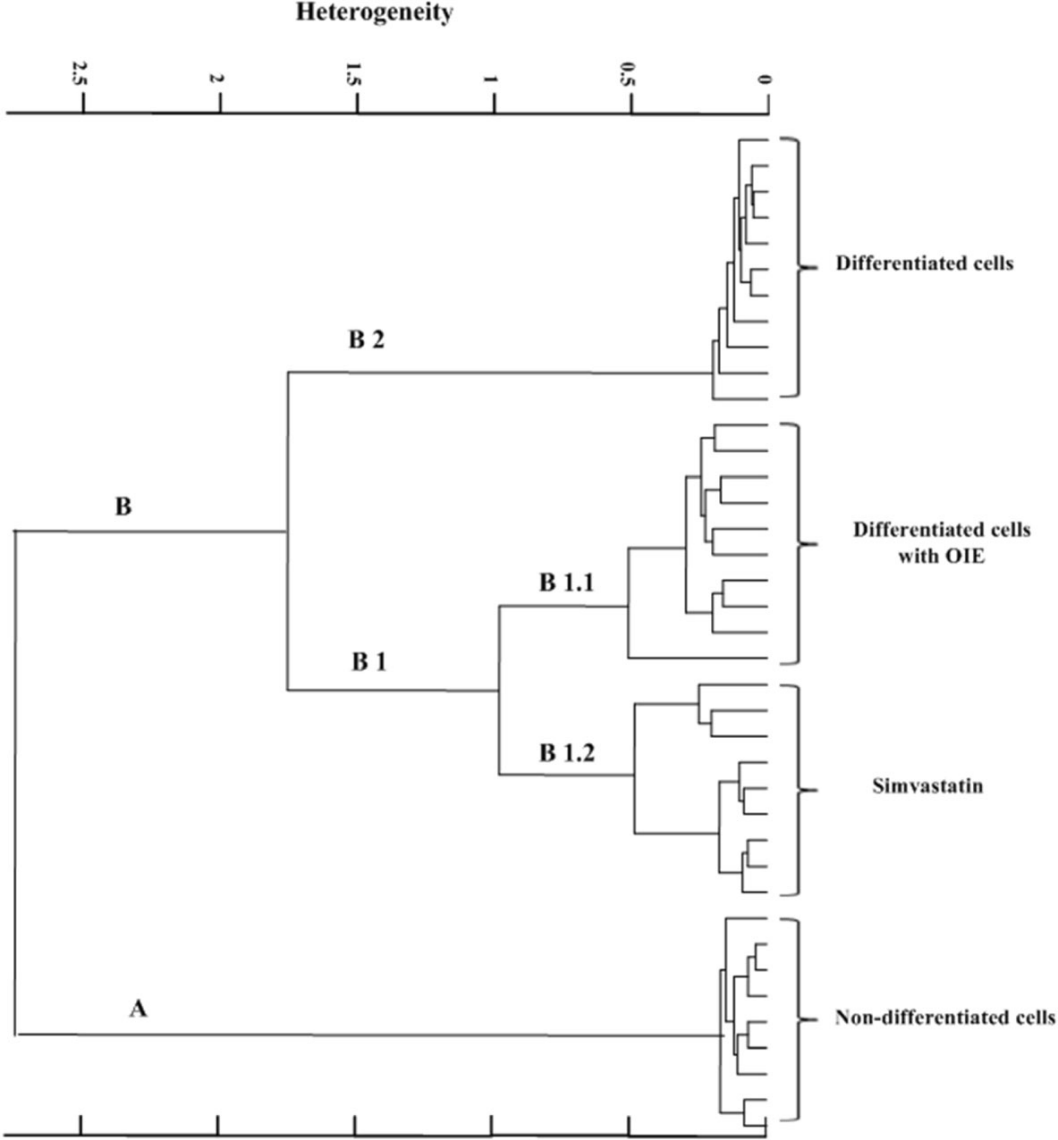
Unsupervised hierarchical cluster analysis (UHCA) dendrogram achieved by cluster classifications of infrared spectra of 3T3-L1 cells. Non-differentiated cells (pre-adipocytes), differentiated cells, simvastatin- and *Oroxylum indicum* extract-treated differentiated cells using second derivative spectra data set. The diagram indicates distinct differences between non-differentiated and differentiated adipocytes. It also demonstrates a difference between OIE treated and non treated adipocytes

Unsupervised Hierarchical Cluster Analysis (UHCA) was used to determine the spectral similarity within groups and between groups of preadipocytes, untreated adipocytes, simvastatin- and OIE-treated adipocytes. The spectral similarity regarding relative distance is illustrated in the dendrogram (Fig. 9), which was obtained using second derivative spectral data and Ward’s algorithm. In simple terms, the lower the heterogeneity, the more similarity exists between the groups. The UHCA analysis indicates that there is a distinct difference between pre-adipocytes and the other cell types. Further to this, the non-treated adipocytes are different from both OIE and simvastatin-treated cells. The results exhibited that the first cluster of pre-adipocyte, branch A, was clearly separated from other groups (branch B). Additionally, the branch B that was corresponding to spectra of untreated adipocytes (branch B2), OIE-treated- (branch B1.1) and simvastatin-treated (branch B1.2) adipocytes was obviously distinguished. The spectra of branch B1 is composed of two subgroups revealing a closer similarity between OIE- and simvastatin-treated adipocytes. Cluster analysis employed Ward’s algorithm using second derivatives, and vector normalization, over the spectral ranges 3000–2800 cm^− 1^ and 1800–950 cm^− 1^. Regarding the result in Fig. 7a, the lipid amount of pre-adipocytes as expected was the lowest (*p* < 0.05).

### Effect of the OIE on pancreatic lipase activity

**Fig. 10:**
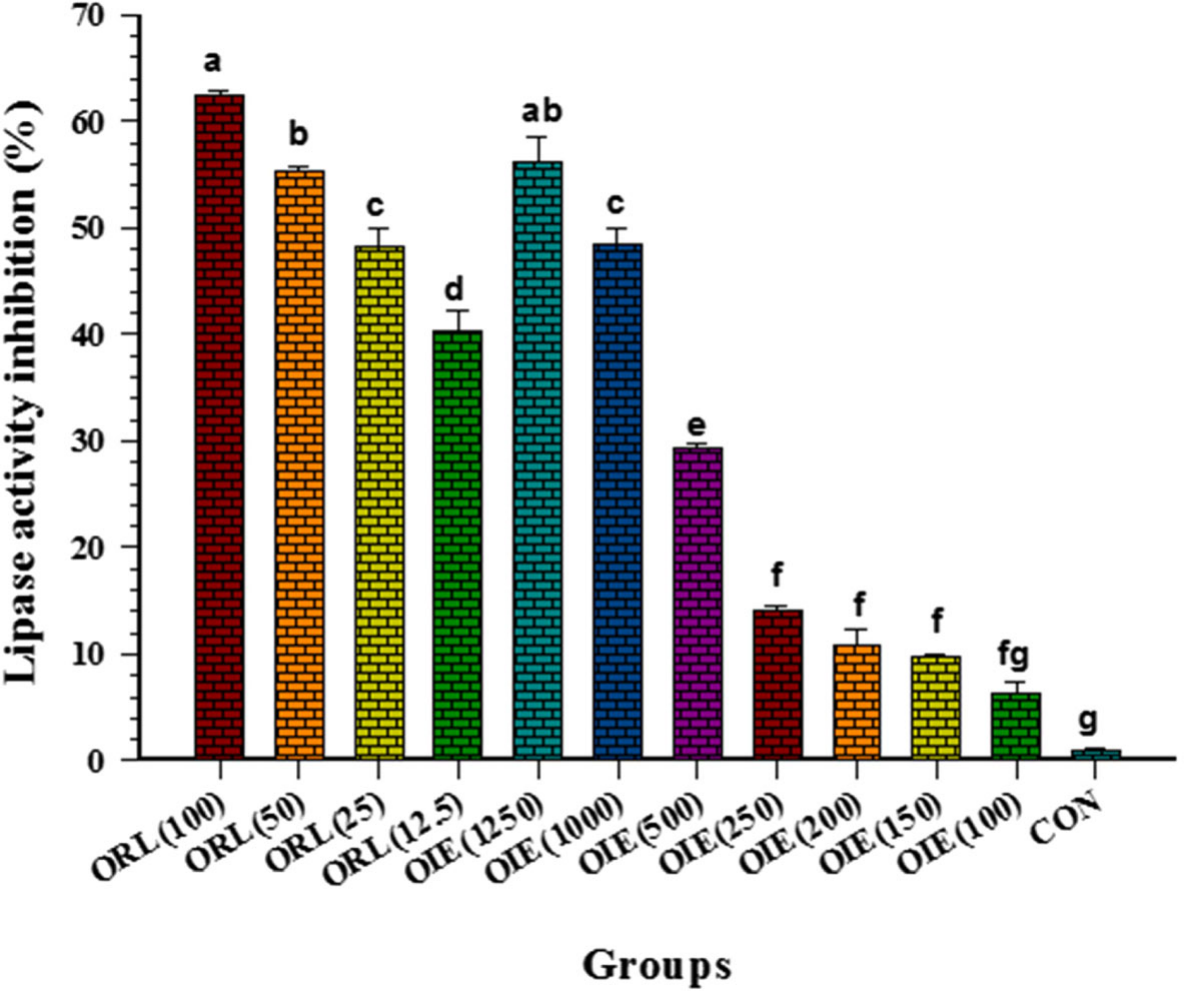
Inhibitory effects of *Oroxylum indicum* extract compare to Orlistat on lipase activity. CON = Control; OIE(100) = *Oroxylum indicum* at 100 μg/mL; ORL(12.5) = Orlistat at 12.5 μg/mL. Orlistat was used as a positive control. Values are represented as Means ± SEM (n = 3). A significant differences were observed (Tukey’s HSD test, *p* < 0.05) and are represented on the figure with letters from a to g. Data suggests that Orlistat is 27 times more effective as inhibitor of lipase compared to OIE

Pancreatic lipase is an enzyme responsible for the hydrolysis of lipid into free fatty acids and glycerol. OIE concentrations between 100 to 1250 μg/mL displayed significantly higher inhibitory lipase activity than those of the controls (*p* < 0.05) (Fig. 10). Moreover, the IC_50_ of OIE for the inhibition of pancreatic lipase was 1062.04 ± 32.21 μg/mL. Whilst the inhibitory effect of the positive control, orlistat at 12.5 to 100 μg/mL, demonstrated an IC_50_ at 38.78 ± 9.55 μg/mL. Under those circumstances, the potential strength of orlistat on lipase activity inhibition is approximately 27 times greater than the OIE. These results suggest that the inhibition of pancreatic lipase activity by OIE increased in a dose-dependent manner.

## Discussion

Adipocyte formation and activity appears to play a central role in the development of obesity. The generation and metabolism of adipocytes have become major targets for treating obesity [34]. One area that has increased is the use of natural products to target obesity. Compared to the convenience and cost they are becoming more attractive propositions than synthetic drugs or surgery [35, 36]. In this study, we investigated the effects of an OIE on the anti-adipogenic and biomolecular change in 3T3-L1 cells. Our studies indicated there was a dose-dependent effect of OIE upon the viability of preadipocyte ranging from concentrations of 250 μg/mL to 1500 μg/mL. At lower doses (0–200 μg/mL) there was no significant difference from the control (*p* > 0.05). Although no major impact on the viability of the cells was observed at lower doses, there may be some toxicological changes to the cells. One further observation was that doses of OIE between 50 to 200 μg/mL indicated a dose-dependent decrease of lipid accumulation in the adipogenesis assay (Figs. 3 and 4). One explanation for the reduction of lipid could be that the chemical components of OIE may have a potent effect which inhibits the differentiation of 3T3-L1 preadipocytes. A previous study reported that the fruit of *O. indicum* is rich in flavonoids such as baicalein [37]. At a concentration of 20 μM baicalein can prevent the differentiation of preadipocyte to adipocyte during first 4 days of induction [38]. In fact, there is evidence to support that baicalein inhibits a cell cycle regulator, promoting cell cycle arrest at the G0/G1 phase. It was also reported to suppress the m-TOR signaling pathway leading to an inhibition of adipogenic factors such as *PPARγ* [39]. OIE at 200 μg/mL lipid accumulation was not significantly different to cells treated with 1.67 μg/mL (4 μM) of simvastatin (Fig. 3, *p* > 0.05). This was a very interesting observation as it confirms the observations made by Nicholson et al. They demonstrated that statins, such as pitavastatin and simvastatin at 5 μM could inhibit adipocyte differentiation by blocking *PPARγ* expression and activating *pref-1* expression [40]. These findings provide evidence that the OIE may also have the capacity to inhibit *PPARγ* activity.

To further elucidate the biochemical potential of OIE, an in vitro pancreatic lipase assay was performed as part of this study. Lipase became a target for research groups attempting to prevent obesity or metabolic syndrome [21, 25]. Pancreatic lipase is an enzyme responsible for the breakdown of triglycerides into glycerol and fatty acid in the gastrointestinal tract. When lipase activity is inhibited, triacylglycerol cannot cross the intestinal brush border membrane leading to a decrease in the uptake of lipids into the human body. Orlistat, a known lipase inhibitor is a drug for treating obesity was used in this study as a positive control. The results of this study indicated that OIE demonstrates as inhibition of pancreatic lipase between doses of 100–1250 μg/mL (IC_50_ of 1062.04 ± 32.21 μg/mL), 27 times less potent than orlistat. The dose of OIE that inhibited pancreatic lipase was also a concentration which induced a decrease in viability of pre-adipocytes (IC_50_ of 882.68 ± 47.99 μg/mL). However, a study by Roh and Jung indicated a number of plant extracts could inhibit pancreatic lipase but had a very little effect on the viability of 3T3-L1 cells [41]. In the light of this publication, it would appear that OIE is not the most effective inhibitor of lipase activity. However, the potential for OIE to inhibit cell cycle progression [39] or its effects on key adipogenic biochemical pathways [16] still requires further investigation.

Although FTIR microspectroscopy has previously been used to characterise biochemical composition in medical research studies [29, 42, 43]. This is the first study using FTIR to demonstrate on the biochemical profile of OIE treated adipocyte. These results indicated that the lipids, lipid esters, nucleic acids, glycogen and carbohydrates of the OIE-treated adipocytes were significantly decreased compared to the untreated adipocytes (Fig. 7a and b). This study indicates that FTIR provided data similar to that obtained using established biochemical assays. This suggests that FTIR is a very useful technique for assessing the impact of plant extracts on established cell lines. Although FTIR supported the preliminary evidence regarding to biochemical changes during the differentiation of 3 T3-L1 cells more research is needed to clarify the mechanism of action of OIE.

## Conclusions

This study suggests that the OIE derived from the fruit pods of the plant inhibited both adipogenesis and lipid accumulation in 3T3-L1 adipocytes. The extract inhibited lipase activity, but this was not as effective as orlistat. FTIR microspectroscopy provides valuable information which supported the biochemical assays used to assess 3T3-L1 cells. The precise mechanism of inhibitory effect on adipogenesis and lipid accumulation is of biochemical interest and requires further investigation.

## Abbreviations

ANOVA: One-way analysis of variance
DMEM: Dulbecco’s modified Eagle’s medium with high glucose
DMSO: Dimethyl sulphoxide
EMSC: Extended multiplicative signal correction
FTIR: Fourier transform-infrared
IBMX: 3-Isobutyl-1-methylxanthine
MTT: 3-(4,5-Dimethylthiazol-2-yl)-2,5-diphenyltetrazolium bromide
OIE: *Oroxylum indicum* extract
PCA: Principal Component Analysis
SEM: The standard error of the mean
TFC: Total flavonoids content
TPC: Total phenolic content
UHCA: Unsupervised Hierarchical Cluster Analysis
